# Landmark- vs Ultrasound-Guided Intercostobrachial Nerve Block and Serratus Plane Block after Supraclavicular Plexus Block for Medial Arm AV Fistula Surgery: A Randomized Double-Blind Trial

**DOI:** 10.5812/aapm-164793

**Published:** 2025-09-13

**Authors:** Mohamed Zakarea Wfa, Hani Gharib Ali, Esraa Hassan Abd Elwahab, Mohamed Ibrahim Adel Eleissawy, Ahmed Kamal Taha

**Affiliations:** 1 Anesthesiology, Surgical Intensive Care and Pain Medicine Department, Faculty of Medicine, Tanta University, Tanta, Egypt; 2 Anesthesia, ICU, and Pain Management, Faculty of Medicine, Mansoura University, Mansoura, Egypt; 3 Vascular and Endovascular Surgery Department, Faculty of Medicine, Tanta University, Tanta, Egypt

**Keywords:** Anesthesia, Arteriovenous Fistula, Supraclavicular Plexus Block, Intercostobrachial, Serratus Anterior Plane Block

## Abstract

**Background:**

Effective postoperative pain management is necessary to enhance patient recovery and satisfaction following the creation of an arteriovenous fistula (AVF).

**Objectives:**

This work compares the role of Ultrasound (US) guided and landmark-guided Intercostobrachial nerve (ICBN) block and serratus plane block (SPB) after supraclavicular plexus block (SCPB) for anesthesia in the creation of AVF in the medial side of the arm.

**Methods:**

This randomized, double-blind trial was carried out on 75 patients, 18 - 65 years old, both sexes undergoing creation AVF in the medial side of the arm. Participants were randomized equally into three groups receiving SCPB, followed by traditional landmark ICBN (TICBN) in group T, US-guided ICBN in group U, or US-guided SPB in group S.

**Results:**

Groups U and S needed less local anesthesia supplementation than group T (8%, 12%, 44%, P < 0.05). Time for first rescue analgesia was delayed in U and S as opposed to T, and in U as opposed to S (P < 0.001). Fentanyl consumption was diminished in U and S than T, and in U than S (P < 0.001). Visual Analogue Scale scores were diminished in U and S as opposed to T at 2 and 4 hours, with no difference between U and S; at 8 hours, T and U had diminished VAS than S (P < 0.05). Patient satisfaction was better in the U than in the T and S (P = 0.002).

**Conclusions:**

US guided ICBN and SPB provide superior anesthesia and postoperative analgesia as opposed to TICBN following the creation of AVF in the arm medial side.

## 1. Background

The Brachial Plexus Block (BPB) is frequently employed in creating proximal arm arteriovenous fistula (AVF) (1). The technique employed for BPB in upper limb surgeries varies depending on the Brachial Plexus availability, with options including neurostimulation, Ultrasound (US), and trans-arterial methods (2). Supraclavicular brachial plexus block (SCPB) is an alternative to traditional BPB techniques, offering comparable anesthesia and postoperative analgesia with a diminished adverse events incidence (3). However, SCPB does not afford complete anesthesia to the arm medial side, which is innervated by the intercostobrachial nerve (ICBN) and the brachial cutaneous nerve medial branch (2). As a result, local anesthetic (LA) supplementation intraoperatively may be necessary to avoid switching to general anesthesia (4). The ICBN can be blocked using two methods: (1) LA can be injected along the nerve pathway for selective ICBN blockade using US guidance; or (2) by relying on the superficial anatomy of the nerve for precise placement (5). Using US guidance, ICBN can be recognized and blocked separately or in combination with other nerves (6). Ultrasound -guided ICBN blockade has been proposed as a logical solution for controlling pain induced by the closure of the tourniquet in the upper extremities (7). The serratus plane block (SPB) is a regional anesthesia approach primarily employed in the thoracic region (8). By targeting the thoracic intercostal nerves, SPB provides adequate anesthesia as well as analgesia for the hemithorax, posterior shoulder, and axillary region (9). We hypothesized that US-guided ICNB and SPB would provide superior postoperative analgesia as opposed to traditional landmark-guided ICNB following SCPB for AVF creation in the medial side of the arm.

## 2. Objectives

The present work compared the role of US-and landmark-guided ICBN and SPB when added after SCPB to determine which technique most effectively covers the medial side of the arm, an area often spared by SCPB alone, during AVF creation.

## 3. Methods

This randomized, double-blind trial was performed on 75 patients, both sexes, aged 18 - 65 years, with physical status classified as III according to the American Society of Anesthesiology and experiencing creation of an AVF in the arm’s medial side. This research was approved by the Institutional Ethics Committee (NO: 36264PR77/5/24) between July 2024 and December 2024. This investigation adhered strictly to the ethical standards set forth by the Declaration of Helsinki. Every participant granted written informed consent before joining the study. In keeping with transparency and regulatory best practices, the trial was formally registered on ClinicalTrials.gov prior to enrolling any patients (NCT06500572). Participants were excluded if they had drug allergies, a Body Mass Index of 35 kg/m² or more, coagulation abnormalities, severe heart, kidney, and liver diseases, pregnancy, vasculitis, unstable hemodynamics, upper extremity neuropathy, mental illness, or seizures. Prior to the surgical procedure, all participants underwent medical history taking, clinical examination, and laboratory testing. Furthermore, they were familiarized with the Visual Analog Scale (VAS) for pain assessment to ensure that they could accurately report their level of pain.

### 3.1. Randomization and Blinding

A random allocation process using computer-generated numbers (https://www.randomizer.org/) was employed to ensure the integrity of the research. All participants’ codes were placed in an opaque, closed envelope to maintain blinding. Participants were randomized equally into three groups (1:1:1 ratio) receiving single SCPB followed by either traditional landmark LCBN (TICBN) in group T, US-guided ICBN in group U, or US-guided SPB in group S. To maintain the blinding, both patients and outcome assessors were blind to group assignments. All blocks were performed by a single experienced anesthesiologist not involved in outcome assessment, ensuring consistency and minimizing performance bias. Standard monitoring in this research included temperature probe, ECG, pulse oximetry, and non-invasive blood pressure. The nerve blocks were administered using a US machine (Philips CX50, extreme edition, Amsterdam, the Netherlands) with a linear probe (6 - 12 MHz) under strict aseptic conditions. Lidocaine 1% was injected at the entry site of the needle. After confirming the needle's location with 1 mL of saline solution, 20 mL of bupivacaine (0.25%) was administered.

### 3.2. Supraclavicular Plexus Block Procedure

**Figure 1.**
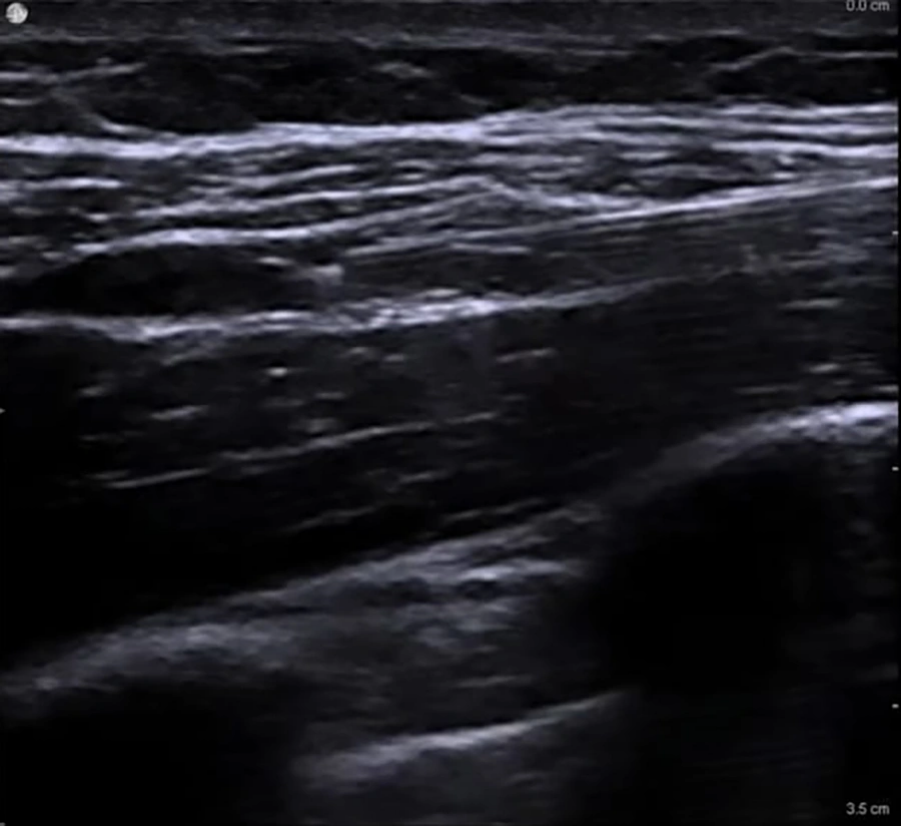
Supraclavicular plexus block (SCPB) procedure

In all patients, the SCPB was performed before the assigned supplemental block (10). Patients were placed supine with the head turned approximately 30° away from the surgical side and a small towel positioned between the scapulae to optimize access. After aseptic preparation, a high-frequency linear US probe (6 - 12 MHz) covered with a sterile sheath was placed in the coronal-oblique plane just superior to the clavicle and lateral to the sternocleidomastoid muscle to visualize the subclavian artery medially, the brachial plexus divisions as a cluster of hypoechoic round structures lateral to the artery, and the first rib and pleura as hyperechoic lines deep to the artery. Using an in-plane lateral-to-medial approach, a 22-G, 80-mm insulated block needle was advanced under continuous US guidance toward the “corner pocket” bordered by the subclavian artery medially, the first rib inferiorly, and the brachial plexus laterally. After negative aspiration, 5 - 10 mL of 0.25% bupivacaine was injected into the corner pocket, and the needle was redirected to deposit additional 3 - 5 mL aliquots around the remaining plexus divisions to ensure circumferential spread, for a total of 20 mL. Proper injectate distribution was confirmed sonographically, and care was taken to avoid pleural puncture, vascular injection, or intraneural spread (Figure 1). 

### 3.3. Traditional Landmark Intercostobrachial Nerve Block Procedure 

In group T, following the SCPB, the intercostobrachial nerve (ICBN) was blocked using a traditional landmark-guided technique (2). The patient was positioned supine with the ipsilateral arm abducted to 90°. The second rib was palpated along the midaxillary line, and the injection point was marked approximately 2 cm inferior to its diminished border. After skin antisepsis, an 80-mm block needle was inserted perpendicular to the skin until gentle contact with the rib was made. The needle was then redirected slightly caudally to slip off the inferior edge, avoiding the intercostal neurovascular bundle. A total of 5 - 8 mL of 0.25% bupivacaine was injected in a fan-shaped manner along the rib, supplemented by subcutaneous infiltration extending anteriorly and posteriorly for 3 - 5 cm to cover the ICBN and its communicating branches.

### 3.4. Ultrasound-Guided Intercostobrachial Nerve Block Procedure 

**Figure 2.**
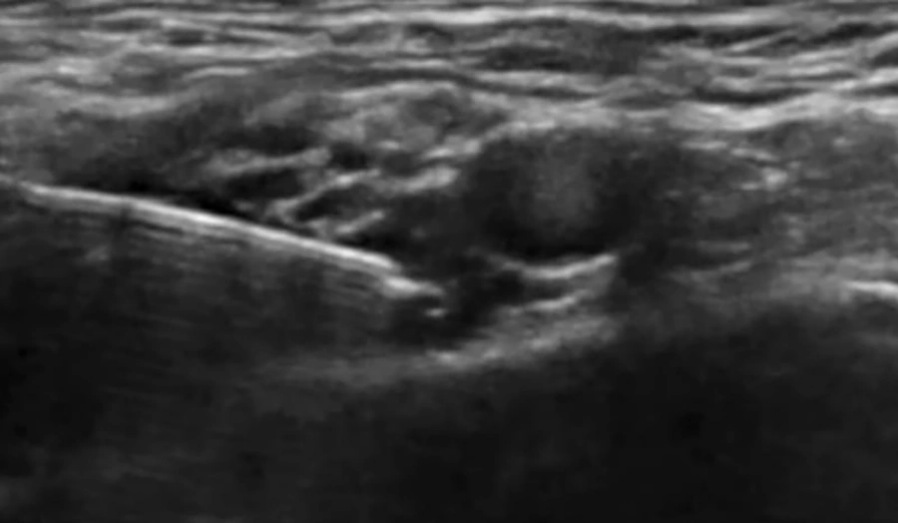
Ultrasound (US) guided intercostobrachial nerve block procedure

In group U, after SCPB, the ICBN was blocked under US guidance at the midaxillary level. The patient was positioned supine with the arm abducted to 90° (11). A high-frequency linear probe (6 - 12 MHz) was placed transversely over the midaxillary line at the level of the 2nd–3rd intercostal spaces. The axillary vein and artery were first visualized, then the probe was adjusted superficially to identify the fascial plane between the subcutaneous tissue and the serratus anterior muscle. The ICBN appeared as a small hyperechoic oval or linear structure within this plane. Using an in-plane, posterior-to-anterior needle approach, an 80-mm block needle was advanced into the fascial plane. After confirming negative aspiration, 5 - 8 mL of 0.25% bupivacaine was slowly injected, and real-time sonographic imaging confirmed the spread of local anesthetic along the plane both anteriorly and posteriorly (Figure 2). 

### 3.5. Serratus Plane Block Procedure 

**Figure 3.**
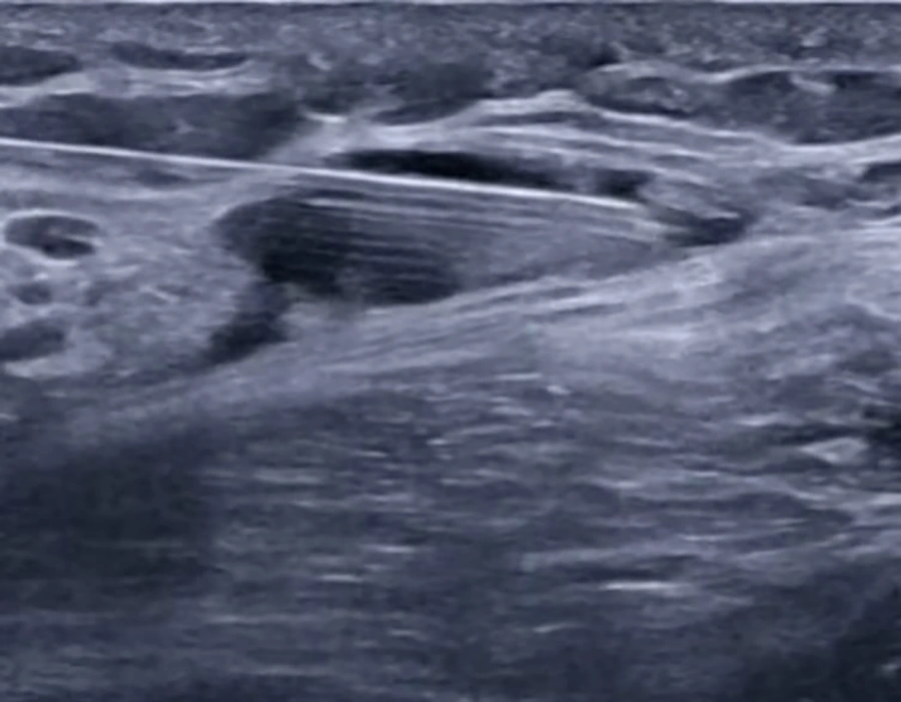
Serratus plane block (SPB) procedure

In group S, following completion of the SCPB, the SAP block was performed with the patient in the lateral decubitus position, operative side up, and the ipsilateral arm abducted to expose the midaxillary region (12). A high-frequency linear US probe (6 - 12 MHz) was positioned over the midaxillary line at the level of the 4th–5th ribs, identifying the latissimus dorsi (LD) muscle superficially and posteriorly, the serratus anterior (SA) muscle deep to LD, and the underlying ribs and pleura. Using an in-plane posterior-to-anterior approach, an 80-mm block needle was advanced under continuous US guidance into the fascial plane between the LD and SA muscles (superficial SAP). After confirming negative aspiration, 20 mL of 0.25% bupivacaine was injected incrementally, with hydrodissection confirming correct placement and real-time imaging verifying spread of local anesthetic along the plane in both cranial and caudal directions to ensure adequate coverage (Figure 3). 

If pain persisted during the operation, repeated local anesthetic infiltration (2% lidocaine, maximum dose 20 mL) was administered. If pain continued after regional anesthesia, the patient was converted to general anesthesia. A standardized postoperative analgesic regimen was implemented. Paracetamol 1 g was administered every 6 hours for patients on regular dialysis and every 8 hours for those not on dialysis. Fentanyl was employed as the rescue analgesic due to its safer profile in renal impairment, administered intravenously in 25 - 50 mcg boluses as needed if the Visual Analog Scale (VAS) score exceeded 3, with repeat doses permitted until the score dropped below 4. Pain was assessed at 0, 2, 4, 8, 12, and 24 hours postoperatively. Adverse effects were monitored, including hypotension (defined as MAP < 65 mmHg or a reduction by 20% as opposed to basal, which was managed with IV fluids; bradycardia (defined as less than 50 beats/min), managed with IV atropine at 0.02 mg/kg; respiratory depression (SpO2 < 95% requiring oxygen supplementation); postoperative nausea and vomiting (PONV), managed with ondansetron at 0.1 mg/kg IV; and any complications related to the nerve block. Patient satisfaction was evaluated using a 5-point Likert scale, where (1) Extremely dissatisfied, (2) unsatisfied, (3) neutral, (4) satisfied, and (5) extremely satisfied (13). The research's primary outcome was the percentage of patients who needed LA supplementation, while secondary outcomes encompassed total fentanyl consumption in the first 24 hours and patient satisfaction.

### 3.6. Sample Size Calculation

The sample size was determined utilizing G*Power 3.1.9.2 (Universität Kiel, Germany). Pilot research was conducted with five cases per group, revealing that 40% of patients needed local anesthesia supplementation with landmark techniques, while 10% required it with US guidance. The determination of the sample size was influenced by several key factors: A 95% confidence limit, 80% power, a 2:1 group ratio, and the inclusion of eight additional cases to accommodate potential dropouts. In total, 75 individuals were enrolled in this research.

### 3.7. Statistical Analysis

The data was analyzed using SPSS v27, which was developed by IBM and is located in Chicago, IL, USA. To check whether the data was distributed normally, we employed histograms and the Shapiro-Wilks test. We employed analysis of variance (ANOVA) with Tukey's post hoc test to examine quantitative parametric data, which was presented as means with standard deviations (SD). Medians with interquartile ranges (IQR) were employed to depict quantitative non-parametric data, and the Kruskal-Wallis and Mann-Whitney tests were employed to compare groups. The Chi-square test was employed to summarize the qualitative variables as frequencies (%). A two-tailed P of less than 0.05 was employed to define statistical significance.

## 4. Results

**Figure 4.**
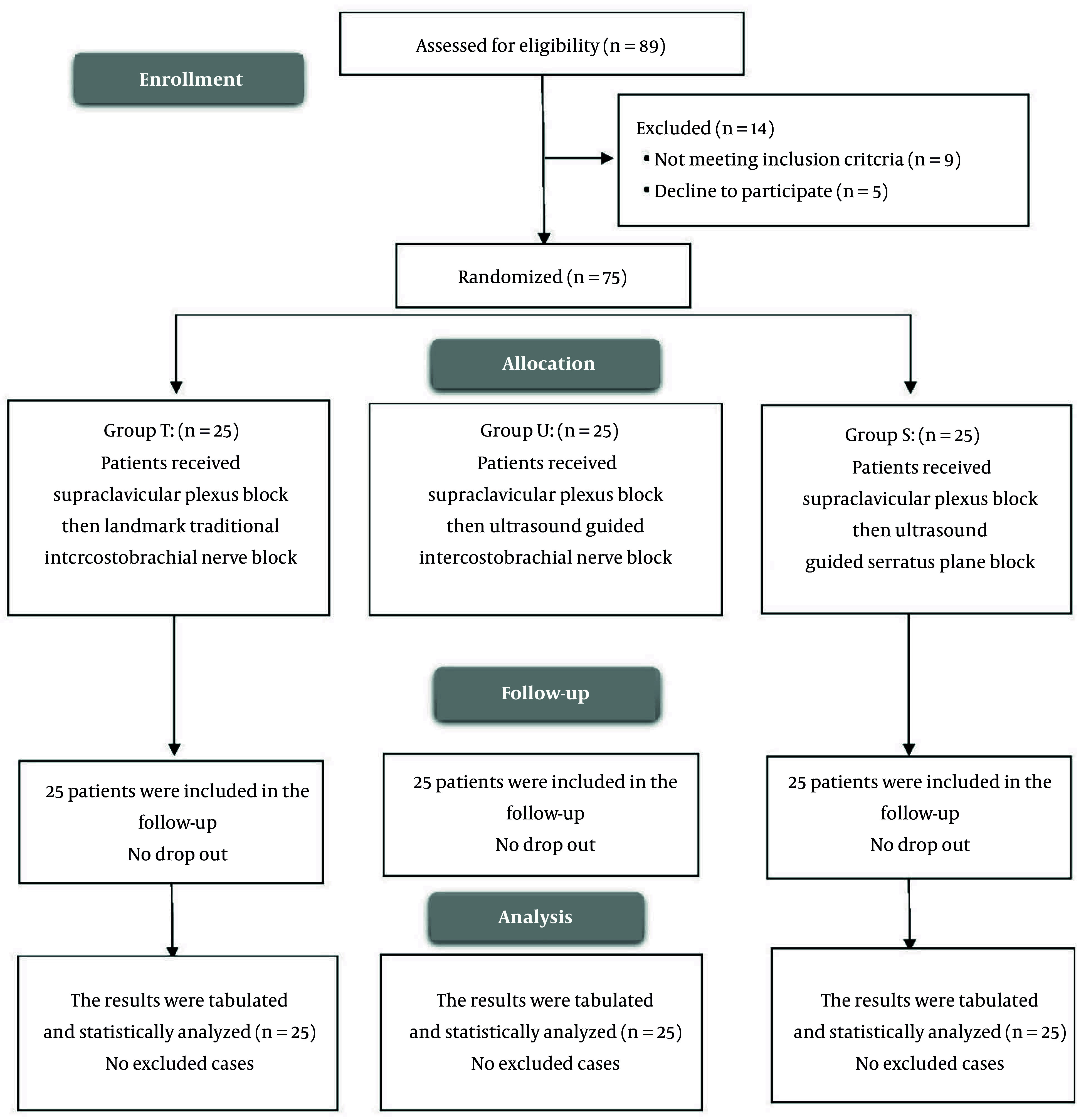
CONSORT flowchart of the enrolled patients

We enrolled 89 cases and evaluated their eligibility for participation. Of these, nine patients did not meet the inclusion criteria, and five declined to participate, leaving 75 patients randomized equally to three groups and followed for analysis (Figure 4). 

Demographic data and surgery duration were comparable among the groups studied (Table 1). 

**Table 1.** Demographic Data and Duration of Surgery of The Studied Groups (N = 25) ^a^

Variables	Group T	Group U	Group S	P-Value
**Age (y)**	42.2 (10.95)	43.7 (10.6)	39.6 (9.7)	0.373
**Sex, n**				
Male/female	15/10	13/12	14 /11	0.850
**Weight (kg)**	77.6 (9.95)	74.4 (7.55)	72.9 (8.78)	0.158
**Height (m)**	1.7 (0.08)	1.68 (0.08)	1.7 (0.07)	0.785
**BMI (kg/m** ^**2**^ **)**	27.2 (4.05)	26.4 (3.66)	25.4 (3.47)	0.250
**ASA physical status (No.)**				
I/II	16 /9	14/10	17 /8	0.780
**Duration of surgery (min)**	83.6 (15.51)	81.6 (16.5)	87.4 (13.78)	0.401

Abbreviations: BMI, Body Mass Index; ASA, American Society of Anesthesiologists.

^a^ Values are presented as mean (SD) unless otherwise indicated.

The proportion of patients requiring LA supplementation was notably reduced in groups U (8%) and S (12%) when compared with group T (44%) (P < 0.05), and the rates in groups U and S were similar. The duration until the first rescue analgesia was notably extended in groups U and S, as opposed to group T, and in group U relative to group S (P < 0.001). Furthermore, the overall fentanyl usage during the first 24 hours was notably reduced in groups U and S as opposed to group T, with group U exhibiting diminished consumption than group S (P < 0.001) (Table 2). 

**Table 2.** Patients Who Needed LA Supplementation, Time of First Rescue Analgesia and Total Fentanyl Consumption in the 1st 24h of the Studied Groups (N = 25) ^a^

**Variables**	**Group T**	**Group U**	**Group S**	**P-Value ** ^**b**^	Post hoc ^b, c^
**Patients who needed LA supplementation (No.)**	11 (44)	2 (8)	3 (12)	0.002	P1 = 0.008, P2 = 0.025, P3 = 1
**Time of first rescue analgesia (h)**	2.9 (0.93)	9.8 (1.46)	7.4 (1.11)	< 0.001	P1 < 0.001, P2 < 0.001, P3 < 0.001
**Total fentanyl consumption in the 1st 24h (mcg)**	59 (15.94)	38 (12.75)	49 (5)	< 0.001	P1 < 0.001, P2 < 0.001, P3 < 0.001

Abbreviation: LA, local anesthesia.

^a^ Values are presented as mean (SD) unless otherwise indicated.

^b^ P < 0.05 is statistically significant.

^c^ P1, P between group I and group II; P2, P between group I and group III; P3, P between group II and group III.

 The VAS scores indicated no significant differences among the groups at 0, 12, and 24 hours. VAS scores were notably reduced by 2 and 4 hours in groups U and S as opposed to group T (P < 0.05), and were similar between groups U and S. At the 8-hour mark, VAS scores were notably reduced in groups T and U when as opposed to group S (P < 0.05), while the scores in groups T and U were like each other (Table 3). 

**Table 3.** VAS Score of the Studied Groups (N = 25) ^a^

Variables	Group T	Group U	Group S	P-Value ^b^	Post hoc ^b, c^
**0h **	1 (1 - 1)	1 (0 - 1)	1 (0 - 1)	0.059	
**2h **	3 (2 - 5)	2 (1 - 3)	2 (2 - 3)	0.002	P1 < 0.001, P2 = 0.022, P3 = 0.270
**4h **	3 (2 - 5)	2 (1 - 3)	2 (1 - 3)	0.012	P1 = 0.008, P2 = 0.013, P3 = 0.879
**8h **	3 (1 - 4)	2 (1 - 4)	3 (3 - 6)	0.002	P1 = 0.333, P2 = 0.013, P3 < 0.001
**12h **	3 (2 - 4)	2 (2 - 4)	3 (2 - 4)	0.207	P1 < 0.001, P2 < 0.921, P3 < 0.001
**24h **	3 (1 - 4)	3 (2 - 4)	3 (2 - 4)	0.810	

Abbreviation: VAS, visual analogue scale.

^a^ Values are presented as median (IQR).

^b^ P < 0.05 is statistically significant.

^c^ P1, P between group I and group II; P2, P between group I and group III; P3, P between group II and group III.

Group U exhibited a significantly higher level of patient satisfaction in comparison to groups T and S (P = 0.002). The occurrences of bradycardia, hypotension, and postoperative nausea and vomiting were comparable across the groups. No instances of pneumothorax, LAST, or respiratory depression were observed in any of the groups (Table 4). 

**Table 4.** Patient Satisfaction and Complications of the Studied Groups (N = 25) ^a^

Variables	Group T	Group U	Group S	P-Values
**Patient satisfaction**				0.002 ^b^
Extremely satisfied	0 (0)	8 (32)	2 (8)	
Satisfied	4 (16)	10 (40)	8 (32)	
Neutral	12 (48)	6 (24)	9 (36)	
Unsatisfied	9 (36)	1 (4)	6 (24)	
Extremely dissatisfied	0 (0)	0 (0)	0 (0)	
**Complications**				
Bradycardia	1 (4)	4 (16)	3 (12)	0.376
Hypotension	2 (8)	6 (24)	4 (16)	0.304
PONV	5 (20)	1 (4)	3 (12)	0.220
Pneumothorax	0 (0)	0 (0)	0 (0)	-
Local anesthetic systemic toxicity	0 (0)	0 (0)	0 (0)	-
Respiratory depression	0 (0)	0 (0)	0 (0)	-

Abbreviation: PONV, postoperative nausea and vomiting.

^a^ Values are presented as No. (%).

^b^ P < 0.05 is statistically significant.

## 5. Discussion

The main observations from this research indicated that the time to 1st rescue analgesia was significantly prolonged in groups U and S as opposed to group T, and longer in group U in contrast with group S. Total fentanyl consumption in the 1st 24 hours was significantly reduced in groups U and S as opposed to group T, and diminished in group U in contrast with group S. The significantly delayed time to first rescue analgesia in the US-guided cohorts (U and S) as opposed to the landmark-guided group (T) further supports the hypothesis that these techniques offer superior pain relief. The choice of a superficial SAPB was based on its anatomical capacity to block the lateral cutaneous branches of the intercostal nerves supplying the medial arm, making it a suitable adjunct to SCPB for AVF creation. Its superficial approach also minimizes the risk of pleural injury (8). Supraclavicular plexus block alone may inadequately cover the medial arm because it spares the ICBN (14, 15). Supplementation with either the ICBN block or the SAPB can improve coverage. To the best of our knowledge, there are currently no published studies directly comparing the role of US-guided and landmark-guided ICBN and SPB following SCPB for anesthesia in AVF creation on the medial side of the arm. In our study, the number of patients requiring LA supplementation was significantly diminished in groups U and S as opposed to group T, with no significant difference between groups U and S. Ultrasound guidance enables more precise nerve localization and potentially more effective blocks than landmark techniques (16), which may contribute to reduced postoperative opioid requirements and improved pain control (17). The SPB has been shown to provide analgesia to the thoracic wall and axillary region, which may overlap with the area innervated by the ICBN (18, 19). Intercostobrachial nerve, performed at the level of the 3rd rib, can achieve complete sensory block in most cases and is effective in reducing the need for general anesthesia, providing faster onset of analgesia, and lowering rescue analgesic requirements (20-22). Intercostobrachial nerve, performed at the level of the 3rd rib, can achieve complete sensory block in most cases and is effective in reducing the need for general anesthesia, providing faster onset of analgesia, and lowering rescue analgesic requirements (23, 24). Our findings are in line with previous studies reporting that US guidance improves the accuracy and efficacy of nerve blocks, leading to better analgesia outcomes, Bhatia and co-authors (25), Chitnis and co-authors (26) and Magazzeni and co-authors (14). Siamdoust and co-authors (15) also demonstrated that ICBN effectively controls tourniquet pain following axillary block, with US guidance increasing block success and safety. Demir and co-authors (9) exhibited that total opioid consumption in the 1st 24h was significantly reduced in group SPB in contrast with the control group. However, Magoon and co-authors (27) found that in post-thoracotomy analgesia in cardiac surgeries, the time of the first rescue analgesia was significantly delayed in group SPB, in contrast with group US-guided ICBN. Total opioid consumption in the first 12h was significantly diminished in group S in comparison with group U. This variation might be related to utilizing a different LA since they gave 2.5 mg/kg of 5% ropivacaine. In our research, VAS was significantly diminished at 2 and 4h in groups U and S, in contrast with group T. The VAS decreased considerably at the eighth grade in groups T and U, in contrast to group S, and showed no significant difference between groups T and U. Similarly, Demir and co-authors (9) found that VAS was significantly decreased in group S in contrast with the control group. Also, Kim et al. (28) noticed that after single-port video-assisted thoracoscopic surgeries, the pain score was comparable between group T and S. Additionally, Öksüz and co-authors (29) demonstrated that VAS scores were recognized to be significantly improved in the S group in comparison to T group. Moreover, patient satisfaction ratings in our research varied significantly, with group U expressing greater satisfaction than groups T and S. The choice of anesthetic technique can influence the overall patient experience. High patient satisfaction is often correlated with effective pain management and reduced reliance on opioid analgesics (30). Therefore, implementing US-guided techniques could enhance patient satisfaction in surgical settings. Demir and co-authors (9) reported the same of our findings as they noted higher levels of patient satisfaction in group S in contrast with the control group. The small sample size and single-center settings limit the research. The research did not assess the impact of the different interventions on functional outcomes, such as range of motion or strength.

### 5.1. Conclusions

Ultrasound-guided ICBN and SPB provide superior anesthesia and postoperative analgesia as opposed to TICBN following the creation of AVF on the arm's medial side as evidenced by diminished number of patients who needed LA supplementation, prolonged time to 1st rescue analgesia, total fentanyl consumption in the 1st 24 hours and diminished pain scores.

## Data Availability

Data are available on reasonable request from the corresponding author.

## Clinical Trial Registration Code:

NCT06500572.

## References

[1] Nofal WH, El Fawal SM, Shoukry AA, Sabek E, Malak W (2017). Ultrasound-guided axillary brachial plexus block versus local infiltration anesthesia for arteriovenous fistula creation at the forearm for hemodialysis in patients with chronic renal failure.. Saudi J Anaesth..

[2] Seyed Siamdoust SA, Zaman B, Noorizad S, Alimian M, Barekati M (2023). Comparison of the Effect of Intercostobrachial Nerve Block with and Without Ultrasound Guidance on Tourniquet Pain After Axillary Block of Brachial Plexus: A Randomized Clinical Trial.. Anesth Pain Med..

[3] Schubert AK, Dinges HC, Wulf H, Wiesmann T (2019). Interscalene versus supraclavicular plexus block for the prevention of postoperative pain after shoulder surgery: A systematic review and meta-analysis.. Eur J Anaesthesiol..

[4] Quek KH, Low EY, Tan YR, Ong ASC, Tang TY, Kam JW (2018). Adding a PECS II block for proximal arm arteriovenous access - a randomised study.. Acta Anaesthesiol Scand..

[5] Feigl G, Aichner E, Mattersberger C, Zahn PK, Avila Gonzalez C, Litz R (2018). Ultrasound-guided anterior approach to the axillary and intercostobrachial nerves in the axillary fossa: an anatomical investigation.. Br J Anaesth..

[6] Munasinghe BM, Subramaniam N, Nimalan S, Sivamayuran P (2021). Ultrasound to the Rescue: Axillary Clearance under Complete Regional Blockade.. Case Rep Anesthesiol..

[7] McLennan L, Haines M, Graham D, Sullivan T, Lawson R, Sivakumar B (2023). Regional Anesthesia in Upper-Limb Surgery.. Ann Plast Surg..

[8] Blanco R, Parras T, McDonnell JG, Prats-Galino A (2013). Serratus plane block: a novel ultrasound-guided thoracic wall nerve block.. Anaesthesia..

[9] Demir U, Yayik AM, Kose M, Aydin ME, Ates I, Ahiskalioglu A (2020). Does the Serratus Plane Block Added to the Interscalene Block Improve the Quality of Anesthesia in Arthroscopic Shoulder Surgery? A Prospective Randomized Study.. Cureus..

[10] Saker WNTM, khalil R, Elhadad MA, Abosakaya AM (2024). Comparison of Supraclavicular Brachial Plexus Block Para-Vascular Approach and Infraclavicular Brachial Plexus Block in providing Surgical Anesthesia for Below Elbow Operation.. Benha J Appl Sci..

[11] Zhu M, Sun W (2024). Application and Research Progress of Ultrasound-Guided Brachial Plexus Block Through Costoclavicular Space Approach in Upper Limb Surgery.. Altern Ther Health Med..

[12] Szamborski M, Janc J, Rosinczuk J, Janc JJ, Lesnik P, Lysenko L (2022). Use of Ultrasound-Guided Interfascial Plane Blocks in Anterior and Lateral Thoracic Wall Region as Safe Method for Patient Anesthesia and Analgesia: Review of Techniques and Approaches during COVID-19 Pandemic.. Int J Environ Res Public Health..

[13] Chen Q, Beal EW, Okunrintemi V, Cerier E, Paredes A, Sun S (2019). The Association Between Patient Satisfaction and Patient-Reported Health Outcomes.. J Patient Exp..

[14] Magazzeni P, Jochum D, Iohom G, Mekler G, Albuisson E, Bouaziz H (2018). Ultrasound-Guided Selective Versus Conventional Block of the Medial Brachial Cutaneous and the Intercostobrachial Nerves: A Randomized Clinical Trial.. Reg Anesth Pain Med..

[15] Sethuraman RM (2025). Regional Anesthesia Techniques for Breast Cancer Surgeries-A Narrative Review.. Indian J Surg Oncol..

[16] Alnaeli GR, Hwisa SA, Ghada MS, Othman R, Fathi, Alkurdi (2024). Comparison between ultrasound and anatomical landmark guided technique for nerve block anesthesia.. NAJSP..

[17] Albrecht E, Chin KJ (2020). Advances in regional anaesthesia and acute pain management: a narrative review.. Anaesthesia..

[18] Oliveira L, Chaves RA (2023). Debridement of axillary necrotizing fasciitis under anesthetic blocks of the serratus plane and supraclavicular brachial plexus: a case report.. Braz J Anesthesiol..

[19] Lee CCM, Beh ZY, Lua CB, Peng K, Fathil SM, Hou JD (2022). Regional Anesthetic and Analgesic Techniques for Clavicle Fractures and Clavicle Surgeries: Part 1-A Scoping Review.. Healthcare (Basel)..

[20] Scholten HJ, Pourtaherian A, Mihajlovic N, Korsten HHM, A. Bouwman R (2017). Improving needle tip identification during ultrasound-guided procedures in anaesthetic practice.. Anaesthesia..

[21] Moustafa MA, Kandeel AA (2018). Randomized comparative study between two different techniques of intercostobrachial nerve block together with brachial plexus block during superficialization of arteriovenous fistula.. J Anesth..

[22] Diakomi M, Maskanakis Α, Makris A, Kouvelos G (2022). B30 The impact of ultrasound guided brachial plexus block on the outcome of arteriovenous fistula creation.. Ultrasound guided RA (UGRA)..

[23] Sanllorente-Sebastian R, Rodriguez-Joris E, Avello-Taboada R, Fernandez-Lopez L, Ayerza-Casas V, Robador-Martinez D (2020). Addition of serratus-intercostal plane block/BRILMA for arteriovenous access surgery.. Rev Esp Anestesiol Reanim (Engl Ed)..

[24] Eskandr A, E Lotfi M, El-Mahrouk RG, A Sultan A (2021). Ultrasound-guided pectoral nerves block type ii or intercostobrachial nerve block as a supplement to supraclavicular block in end-stage renal disease patients' arteriovenous access: A randomized controlled trial.. J Cell Mol Anesth..

[25] Bhatia A, Brull R (2013). Review article: is ultrasound guidance advantageous for interventional pain management? A systematic review of chronic pain outcomes.. Anesth Analg..

[26] Chitnis SS, Tang R, Mariano ER (2020). The role of regional analgesia in personalized postoperative pain management.. Korean J Anesthesiol..

[27] Magoon R, Kaushal B, Chauhan S, Bhoi D, Bisoi AK, Khan MA (2020). A randomised controlled comparison of serratus anterior plane, pectoral nerves and intercostal nerve block for post-thoracotomy analgesia in adult cardiac surgery.. Indian J Anaesth..

[28] Kim S, Bae CM, Do YW, Moon S, Baek SI, Lee DH (2021). Serratus Anterior Plane Block and Intercostal Nerve Block after Thoracoscopic Surgery.. Thorac Cardiovasc Surg..

[29] Öksüz G, Sayan M, Arslan M, URFALIOĞLU A, Öksüz H, Bilal B (2018). The comparison of serratus anterior plane block versus intercostal block for postoperative analgesia following thoracotomy surgery.. J Anesthesia/Anestezi Dergisi (JARSS)..

[30] Glowacki D (2015). Effective pain management and improvements in patients' outcomes and satisfaction.. Crit Care Nurse..

